# Supplementary material for: Building Protein Atomic Models from Cryo-EM Density Maps and Residue Co-Evolution
Source: Biomolecules. 2022 Sep 13;12(9):1290. doi: 10.3390/biom12091290 (PMC9496541; doi:10.3390/biom12091290)
Supplement: Supplementary file 1 [file biomolecules-12-01290-s001.zip › biomolecules-1865128-supplementary.pdf]

Building Protein Atomic Models from Cryo-EM Density Maps and Residue Co-Evolution

Guillaume Bouvier[1,*], Benjamin Bardiaux[1,*], Riccardo Pellarin[1], Chiara Rapisarda[2,3] and Michael Nilges[1]

[1] Institut Pasteur, Université Paris Cité, CNRS UMR 3528, Structural Bioinformatics Unit, 75015 Paris, France

[2] CNRS UMR 5234, Microbiologie Fondamentale et Pathogénicité, University of Bordeaux, 33076 Bordeaux, France

[3] Institut Européen de Chimie et Biologie, University of Bordeaux, 33600 Pessac, France

* Correspondence: guillaume.bouvier@pasteur.fr; benjamin.bardiaux@pasteur.fr

Supplementary Information

Table S1: Additional representative targets

**Target**	**EM Resolution**	**Number of chains (in ASU)**	**Number of amino acids (chain id)**	**Number of predicted contacts (chain id)**	**Number of predicted contacts/residue (chain id)**
3J7H/ EMD-5995	3.20 Å	1	1022 (A)	1450 (A)	1.41
5A63/ EMD-3061	3.40 Å	4	665 (A)<br>215 (B)<br>243 (C)<br>100 (D)<br>(total: 1223)	914 (A)<br>149 (B)<br>156 (C)<br>45 (D)<br>(total: 1264)	1.34 (A)<br>0.69 (B)<br>0.65 (C)<br>0.45 (D)

Table S2: Results of fragmentation and model reconstruction for additional representative targets. Fragments were generated either with the MS Tree presented in this work or from the reference PDB with random fragment length (to evaluate separately the fragmentation and sequence assignment procedure)

Target	Fragment method	Number of fragments	RMSD fragments[(a)]	Coverage fragments[(b)]	RMSD (chain id)	Coverage (chain id) [(c)]
3J7H/ EMD-5995	MS Tree	41	1.2 Å	94 %	47.8 Å (A)	85.9 % (A)
3J7H/ EMD-5995	PDB random length	33	0 Å	100 %	0.9 Å (A)	95.5 % (A)
5A63/ EMD-3061	MS Tree	38	1.46 Å	97 %	5.7 Å (A)<br>29.1 Å (B)<br>4.7 Å (C)<br>41.7 Å (D)	78.8 % (A)<br>40.9 % (B)<br>81.1 % (C)<br>50.0 % (D)
5A63/ EMD-3061	PDB random length	28	0 Å	96.5 %	0.4 Å (A)<br>1.3 Å (B)<br>0.5 Å (C)<br>2.1 Å (D)	90.8 % (A)<br>75.3 % (B)<br>82.7 % (C)<br>84.0 % (D)

(a) RMSD to closest Calpha atom in reference
(a) Percentage of Calpha with distance < 5 Å in reference
(b) Percentage of modeled residues

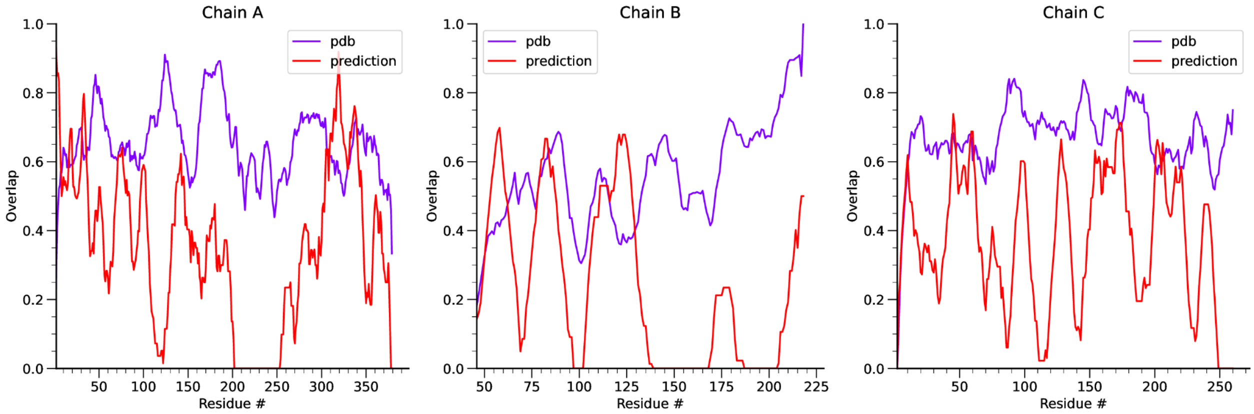

Figure S1: Overlap scores between contact maps of the reference PDB and contact map of the final model (in blue) compared with the overlap scores of the predicted contacts with the final model (in red) for chain A, B and C of 4CI0 PDB model. An overlap of 1 means that all predicted contacts are satisfied by the model. The overlap is computed for sliding fragments of 11 residues along the sequence.

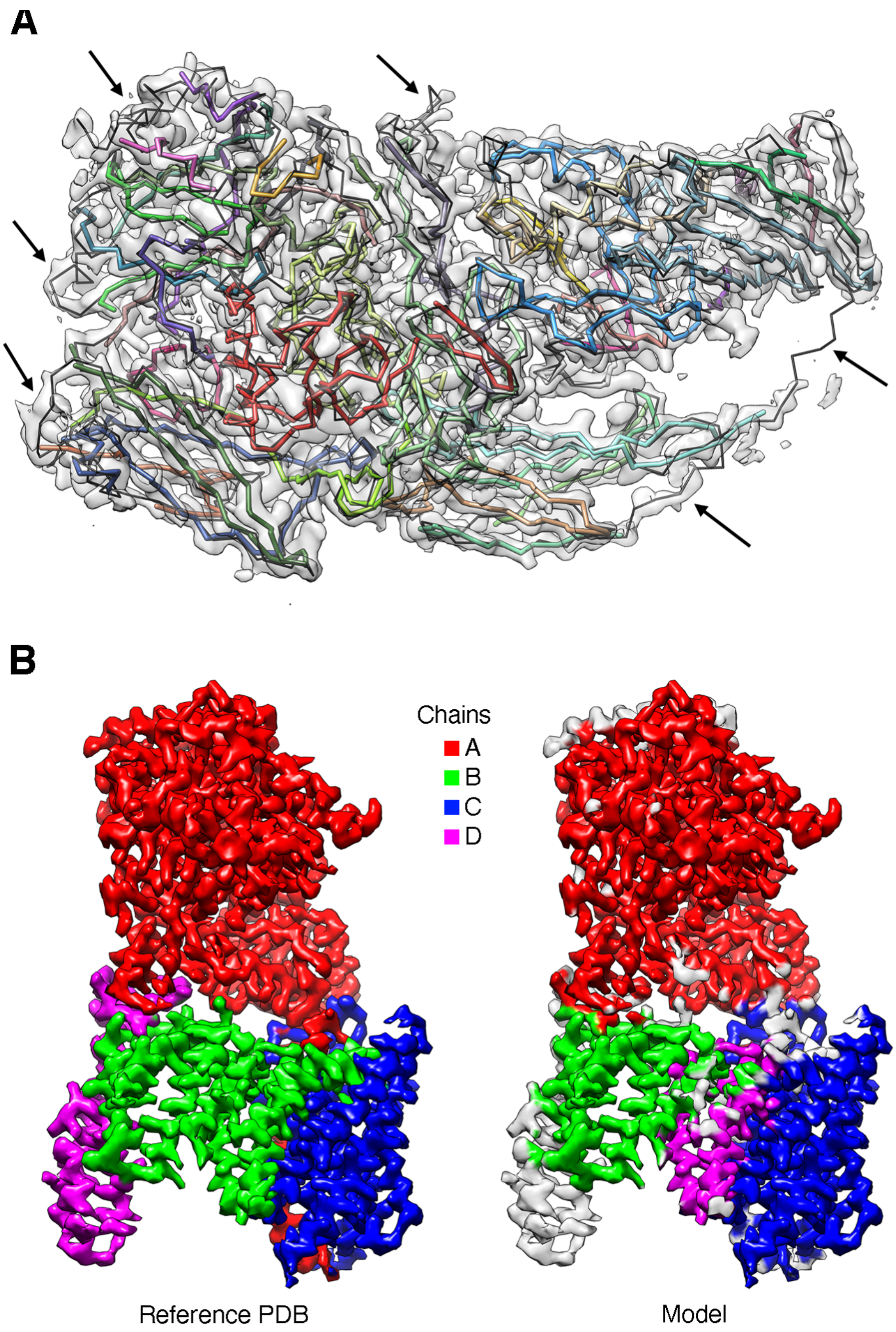

Figure S2: Fragments and segmentation for additional targets. (A) Fragments generated for the 3J7H targets (colored sticks) in the EM density. The Calpha trace of the reference PDB structure is shown black. Regions with breaks in fragment tracing are indicated with arrows. **(B)** Map segmentation for the 5A63 target colored by chains using the atomic coordinates in the reference PDB structure (left) and the reconstructed model using EC (left).

Algorithm S1: Pseudocode for the fragment merging procedure. `f.score` is the alignment score of fragment `f`. `f.neighbors` are the spatial neighbors of fragment `f`. The chain with the maximum alignment score is stored in variable `chain`.

```
S: set of fragments
score_max = -inf
while len(S) > 0:
    f = max(S)
    if f.score > score_max:
        score_max = f.score
        chain = f
    S -= f  # remove fragment f from the set S
    for n in f.neighbors:

       S += merge(f, n)  # add the merged fragment to the set S
```
